# RECIST and CHOI criteria in the evaluation of tumor response in patients with metastatic colorectal cancer treated with regorafenib, a prospective multicenter study

**DOI:** 10.1186/s40644-019-0271-z

**Published:** 2019-12-09

**Authors:** Olivier Lucidarme, Mathilde Wagner, Paul Gillard, Stefano Kim, Jean-Baptiste Bachet, Benoit Rousseau, Thibault Mazard, Christophe Louvet, Benoist Chibaudel, Romain Cohen, Marie-Line Garcia-Larnicol, Aurelien Gobert, Julie Henriques, Thierry André

**Affiliations:** 1 Sorbonne Université and Hôpital Pitié-Salpêtrière, Assistance Publique-Hôpitaux de Paris, CNRS, INSERM, Laboratoire d’Imagerie Biomédicale (LIB), 75013 Paris, France; 2 Hôpital Jean Minjoz, 25030 Besançon, France; 3 Sorbonne Université and Hôpital Pitié Salpêtrière, 75013 Paris, France; 4 GERCOR (Groupe Cooperateur Multidisciplinaire en Oncologie), 151 rue du Faubourg Saint Antoine, 75011 Paris, France; 5 Hôpital Henri Mondor, Assistance Publique-Hôpitaux de Paris, 94010 Créteil, France; 6 ICM, Institut du Cancer de Montpellier, 208 rue des Apothicaires, 34298 Montpellier Cedex 05, France; 7 Institut Mutualiste Montsouris (IMM), 42 Boulevard Jourdan, 75014 Paris, France; 8 Institut Hospitalier Franco-Britannique, Service d’Oncologie Médicale, 4, rue Kléber, 92300 Levallois-Perret, France; 9 Sorbonne Université and Hôpital Saint-Antoine, Assistance Publique-Hôpitaux de Paris, 75012 Paris, France; 10 Sorbonne Université and Hôpital Pitié-Salpêtrière, Assistance Publique-Hôpitaux de Paris, 75013 Paris, France; 11 Methodology and Quality of Life Unit, University Hospital of Besancon, INSERM UMR 1098, 25030 Besançon, France

**Keywords:** Computed tomography, Tumor response, Colorectal cancer

## Abstract

**Background:**

To evaluate the objective response rate (ORR) at 2 months of treatment with regorafenib according to RECIST 1.1, Choi, and modified Choi (mChoi) criteria in patients with metastatic colorectal cancer (mCRC).

**Methods:**

Baseline and 2-month contrast-enhanced computed-tomography (CECT) scans of 55 patients with mCRC, prospectively enrolled in phase II TEXCAN trial, were centrally assessed. The primary endpoint was 2-month ORR by RECIST 1.1, Choi, and mChoi criteria. Final outcome was overall survival (OS).

**Results:**

Of 55 patients included in this study (Intention-to-treat [ITT1] population), 35 had CECT at 2 months (ITT2 population). According to RECIST 1.1 criteria, 20 (57%) patients were SD and 15 were PD (43%) in the ITT2 population. According to Choi criteria, 18 (51%) patients were responders and 17 (48%) were non-responders. Median OS was 5.3 months (95% CI 3.7–8.6) in the ITT1 population and 8.9 months (95% CI 5.1–12.6) in the ITT2 population. In the ITT2 population, median OS was 16 months (95% CI 6.6–17.5) in SD patients (*n* = 20) and 4.6 months (95% CI 3.3–5.8) in PD patients (*n* = 15), according to RECIST 1.1 criteria (HR = 6.48). Median OS was 7.9 months (95% CI 4.2–17.5) in responders (*n* = 18) and 9.9 months (95% CI 3.7-NA) in non-responders (*n* = 17) according to Choi criteria (HR = 1.06). All patients except one were classified as non-responders with mChoi criteria.

**Conclusion:**

At 2 months, unlike RECIST 1.1, Choi and mChoi criteria could not identify mCRC patients with regorafenib survival benefit.

**Trial registration:**

ClinicalTrials.gov Identifier: NCT02699073.Registered March 4, 2016, Retrospectively registered.

## Key points

- At 2 months, Choi and mChoi criteria were not able to identify mCRC patients with a regorafenib OS benefit.
- RECIST 1.1 criteria identify survival benefit at 2 months despite the absence of partial responders with these criteria.

## Background

Until now, the Response Evaluation Criteria in Solid Tumors (RECIST) based on anatomic measurement of the tumor size have been the most widely used for tumor imaging in drug trials or in clinical practice. These criteria provide standardized assessment of the tumor response in terms of progression-free survival (PFS) or time to progression, considered acceptable surrogate endpoints for overall survival (OS) [1]. However, traditional RECIST usually evaluate the responses late and are of limited use as primary endpoint in clinical trials evaluating targeted therapies, especially those with an antiangiogenic and/or an anti-proliferative effect. The ability to differentiate between response or stable disease (SD) and progressive disease (PD) early during therapy is crucial for allowing adjustment of therapy. This is the reason why Choi et al. proposed to take into account not only the size of the tumor, but also drug-induced necrosis [2]. These new response criteria combined a change in tumor size (10% decrease of the largest diameter regardless the attenuation change) or tumor attenuation (15% decrease in Hounsfield units [HU] regardless the size change) on contrast-enhanced computed-tomography (CECT) scans. Choi criteria correlated better than RECIST with disease-specific survival of imatinib-treated gastrointestinal stromal tumors (GIST) patients [3]. Given that tyrosine kinase inhibitor (TKI) can induce necrosis, in metastatic renal cell carcinomas (mRCCs) but little change in size, some authors proposed to extend Choi criteria to sunitinib-treated mRCCs [3, 4]. They found that predictive PFS and OS values of Choi criteria measured during the portal phase at 2 months were significantly better than RECIST 1.0 criteria. However, this finding was not confirmed in two other studies [5, 6] whose authors concluded that neither RECIST nor standard Choi criteria successfully discriminate between TKI-treated mRCC patients with short versus long-term clinical benefit. Nathan et al. [6] proposed using modified Choi (mChoi) criteria, which require a 10% size decrease and a 15% attenuation decrease to define a partial response (PR), while others [7, 8] proposed using only a 10% decrease of the longest diameter as a threshold for classifying PR. Those two last criteria seemed to correspond better with time to progression than RECIST and standard Choi criteria in mRCC patients treated with TKI [6]. Choi criteria appeared more appropriate than RECIST 1.1 to identify responders with long survival among advanced hepatocellular carcinoma patients benefiting from sorafenib [9]. The study of mCRC evaluating an optimal morphologic response (based on qualitative assessment of necrosis) to preoperative chemotherapy with bevacizumab, also supported the potential use of Choi or mChoi criteria [10].

Among new targeted therapies, regorafenib, an oral agent that blocks multiple protein kinases, demonstrated significant clinical efficacy in mCRC patients. In an international pivotal phase III CORRECT study of refractory or advanced mCRC, patients with progression during or within 3 months of the last standard therapy showed both PFS and OS benefit compared to placebo [11]. Median OS was 6.4 months in the regorafenib arm versus 5 months in the placebo arm (HR 0.77; 95% CI 0·64–0·94; one-sided *p* = 0.0052). It is possible that a potential subpopulation of patients with mCRC can benefit more from regorafenib therapy. Hence, a more appropriate selection of patients and identification of predictors of early clinical benefit would be useful.

the objective of this study was to evaluate RECIST1.1, CHOI, mCHOI, or RECIST modified with a 10% decrease of the longest diameter as a threshold for classifying PR (RECIST10%) criteria to assess which provides the best early response assessment to regorafenib in patients with mCRC.

## Materials and methods

### Patients

A total of 55 patient with mCRC who had been treated with regorafenib after a fluoropyrimide-based, an anti-vascular endothelial growth factor (VEGF) and an anti-epidermal growth factor receptor (EGFR) therapy in prospective, open-label, single-arm phase II TEXCAN trial in seven French centers from February 2016 to June 2017, were analyzed. Patients were treated until progression or unacceptable adverse events. Main inclusion criteria were i) disease progression in patients with histologically proven mCRC, who had been previously treated with, or were not considered candidates for, available therapies including fluoropyrimidine-based chemotherapy, ii) an anti-VEGF and an anti-EGFR therapy (if patients were KRAS wild-type), iii) Eastern Cooperative Oncology Group Performance Status (ECOG PS) 0 or 1, iv) at least one target lesion on CT scan, and v) adequate renal, hematological and liver functions. The initial and maximal dose of regorafenib was 160 mg (4 tablets of 40 mg) taken once daily for 3 weeks followed by 1 week off therapy. Dose interruptions and/or reductions were required based on individual safety. Patients continued regorafenib until PD according to RECIST, unacceptable toxic effects, withdrawal of patient’s consent, decision of discontinuation taken by the investigator in the patient’s best interest, or death. If PD according to RECIST criteria, but tumor response by CHOI criteria, the patient continued regorafenib at the investigator’s discretion in case of evident clinical benefit.

### Imaging acquisition

Thoracic–abdominal–pelvic CECT scans after non-standardized intravenous (IV) injection of a low-osmolar, non-ionic contrast agent with an iodine concentration of 300–350 mg/mL at 2 mL/sec were collected. Two acquisition protocols were used according to each center’s usual procedures. For each patient, the same acquisition protocol was used at baseline and follow-up CECT. Thoracic–abdominal–pelvic CECT images for 32 patients were obtained in one acquisition during the portal phase (70–90 s post-injection). Thorax CECT images were obtained during the arterial phase of enhancement (25 s post-injection), while abdomen and pelvis (from the dome of the diaphragm to the pubis) CECT images were acquired during the portal phase (70–90 s post-injection) for the other 23 patients. Multidetector computed tomography (MDCT) parameters were: tube current 150 mAs, tube voltage 120 Kv, detector collimation of 2–3 mm.

Images were taken by the picture archiving and communication systems (PACS) system in each center. An anonymized copy of the exam was collected by the center in charge of the central reading (Radiology Department of La Pitié Salpêtrière Hospital, Paris).

### Imaging analysis

A central radiological review for imaging interpretation was composed by two radiologists with 25 and 4 years, respectively, of experience in the evaluation of abdominal imaging. Radiologists performed a consensus reading of all CECT scans and were blinded to the patients’ clinical data and outcome.

First, only the baseline CECT was displayed on a workstation (Myrian® 1.13.1, Intrasense, Paris, France) to perform the target lesions selection. A maximum of five (two per organ) target lesions >10 mm for the largest diameter were selected for being representative of all involved organs as recommended by RECIST 1.1 [12]. The largest axial diameter of each target lesion was measured manually with calipers, except for the lymph nodes whose small diameters were measured. The attenuation values in Hounsfield units (HU) were obtained in free-hand region of interest (ROI) drawn about 1 mm inside the lesion boundaries on the CECT section whose largest diameter had been measured. For lung lesions, a narrow window was used to avoid inclusion of voxels containing air in the measured area. Then, follow-up CECT images were also displayed on the workstation and were compared to baseline scans to achieve rigorous concordance in the identification of all target lesions during follow-up. The largest axial diameter and the attenuation measurements were recorded as described above.

### Data analysis

For each patient, the sum of all the largest axial diameters was computed and the averages of mean and median attenuations in ROI images of all target lesions were calculated. Absolute and percent change between baseline and the 2-month evaluation was calculated for each patient. Tumor response was assessed according to RECIST 1.1, Choi, mChoi, and RECIST_10%_ (Table 1). For evaluation by RECIST 1.1, responders had complete response (CR) and partial response (PR). CR, PR, and SD were considered disease controlled responses. For evaluation by Choi criteria, responders were defined as having ≥10% decrease of the sum of the largest tumor diameter or ≥ 15% decrease of the mean attenuation computed for the ROIs of all target lesions. For evaluation by mChoi criteria, responders were defined as having ≥10% decrease of sum of the largest tumor diameter and ≥ 15% decrease of mean attenuation computed for the ROIs of all target lesions. For evaluation by RECIST_10%_, responders had CR and PR defined by a − 10% of the longest diameter threshold. Patients who did not meet the RECIST 1.1, Choi, mChoi, and RECIST _10_ response criteria were considered non-responders.

**Table 1:** summary of the response criteria used in the study

	RECIST 1.1	RECIST 10%	CHOI	mCHOI
Measurement	largest Diameter	largest diameter	largest diameter + attenuation^a^	largest diameter + attenuation^a^
Responders	Decrease ≥30% of the sum of LD of TL or complete response	Decrease >10% of the sum of LD of TL or complete response	Decrease ≥10% of the sum of LD of TL or ≥ 15% of the mean attenuation of TL	Decrease ≥10% of the sum of LD of TL and ≥ 15% of the mean attenuation of TL
Non responders	Do not meet the responder criteria			

^a^attenuation in Hounsfield units assessed in a free-hand region of interest drawn about 1 mm inside the lesion boundaries on the CECT section whose largest diameter had been measured

*LD* largest diameter, *TL* Target lesions, *CECT* Contrast enhanced computed tomography

The primary endpoint was the tumor response rate (ORR) at 2 months according to RECIST 1.1, Choi, mChoi, and RECIST_10%_ criteria. Final outcome was OS.

### Statistical analyses

The primary objective of this study was to evaluate which of criteria, RECIST1.1, Choi, provide the best early (at 2 months) response assessment to regorafenib treatment in patients receiving regorafenib for mCRC after standard therapy. Secondary objective were also to evaluate mChoi and RECIST_10%_ at 2 months.

The primary population for efficacy analyses was the intention-to-treat population (ITT1), which was defined as all patients having received at least one tablet of study drug. For the primary endpoint evaluation it was the population defined as patients who underwent CT scans at baseline and at 2 months (ITT2). Primary endpoint was described using percent with 95% confidence intervals (CI). OS was measured from the administration date of regorafenib to the date of death, regardless of the cause, or censored at the time of the last follow-up visit. Survival curves were prepared using the Kaplan-Meier method and were compared using the log-rank Mantel-Cox test in accordance with the final response outcomes. Cox proportional hazards regression model was used to compare survival according to radiological responses using Choi, mChoi, RECIST 1.1, and RECIST_10%_. All statistical tests were two-tailed. A *p* value of .05 was considered significant and 95% CI were calculated. A Fisher’s exact test was used for comparison of frequency. The results of safety analysis will be published in a separate paper.

## Results

**Fig. 1:**
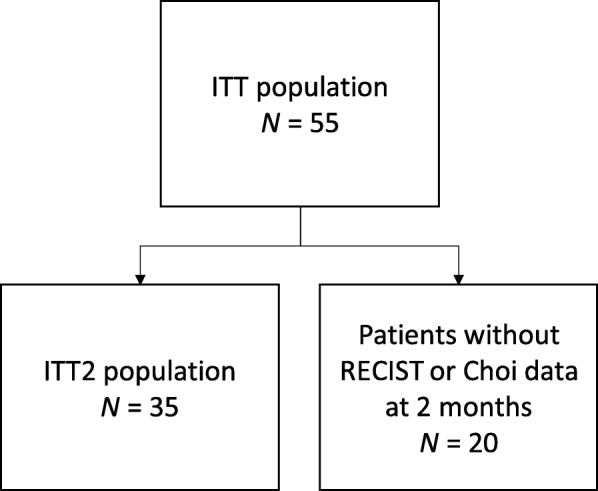
Study flow chart

The study flow chart is presented in Fig. 1. Fitty-five patients were included in the study and all received at least one tablet of regorafenib (ITT1 population). Twenty patients were not reevaluated at 2 months because of RECIST or clinical, biological progression at 1 month (*n* = 7), absence of CECT at 2 months (*n* = 5), death *(n* = 4), adverse event (*n* = 3), or consent withdrawal (*n* = 1). Therefore, the primary endpoint was assessed in 35 patients (ITT2 population). Patient and tumor characteristics are given in Table 2.

**Table 2:** Patient and tumor characteristics

		ITT *N* = 55	ITT2 *N* = 35
		*n* %	*n* %
Gender	Male	30 (55)	22 (63)
	Female	25 (45)	13 (37)
Age	Mean	61	61
	SD	10.5	9.4
	Range	53–69	51–69
Primary tumor present at baseline		16 (29)	12 (34)
ECOG PS	0 vs 1	55 (100)	35 (100)
Stage at initial diagnosis	I	1 (2)	1 (3)
	II	2 (4)	0
	III	12 (22)	8 (23)
	IV	40 (73)	26 (74)
Number of site (s) involved	1	12 (22)	8 (23)
	2	20 (36)	15 (43)
	>2	23 (42)	12 (34)
Prior therapies received			
Fluoropyrimidines		55 (100)	35 (100)
Oxaliplatin		53 (96)	35 (100)
Irinotecan		55 (100)	35 (100)
VEGF inhibitors^a^		53 (96)	34 (97)
EGFR inhibitors^b^		27 (49)	20 (57)

Abbreviations: *SD* Standard deviation, *ECOG PS* Eastern Cooperative Oncology Group Performance Status, *EGFR* Epidermal growth factor receptor, *VEGF* Vascular endothelial growth factor

^a^VEGF bevacizumab and aflibercept

^b^EGFR cetuximab and panitumumab

Seventy five target lesions were identified and studied in the ITT2 population. Overall, four (11%) patients had only one target lesion, 25 (71%) had two, three (8%) had three, three (8%) had four, and none (3%) had five for a mean of 2.1 target lesions per patient.

**Fig. 2:**
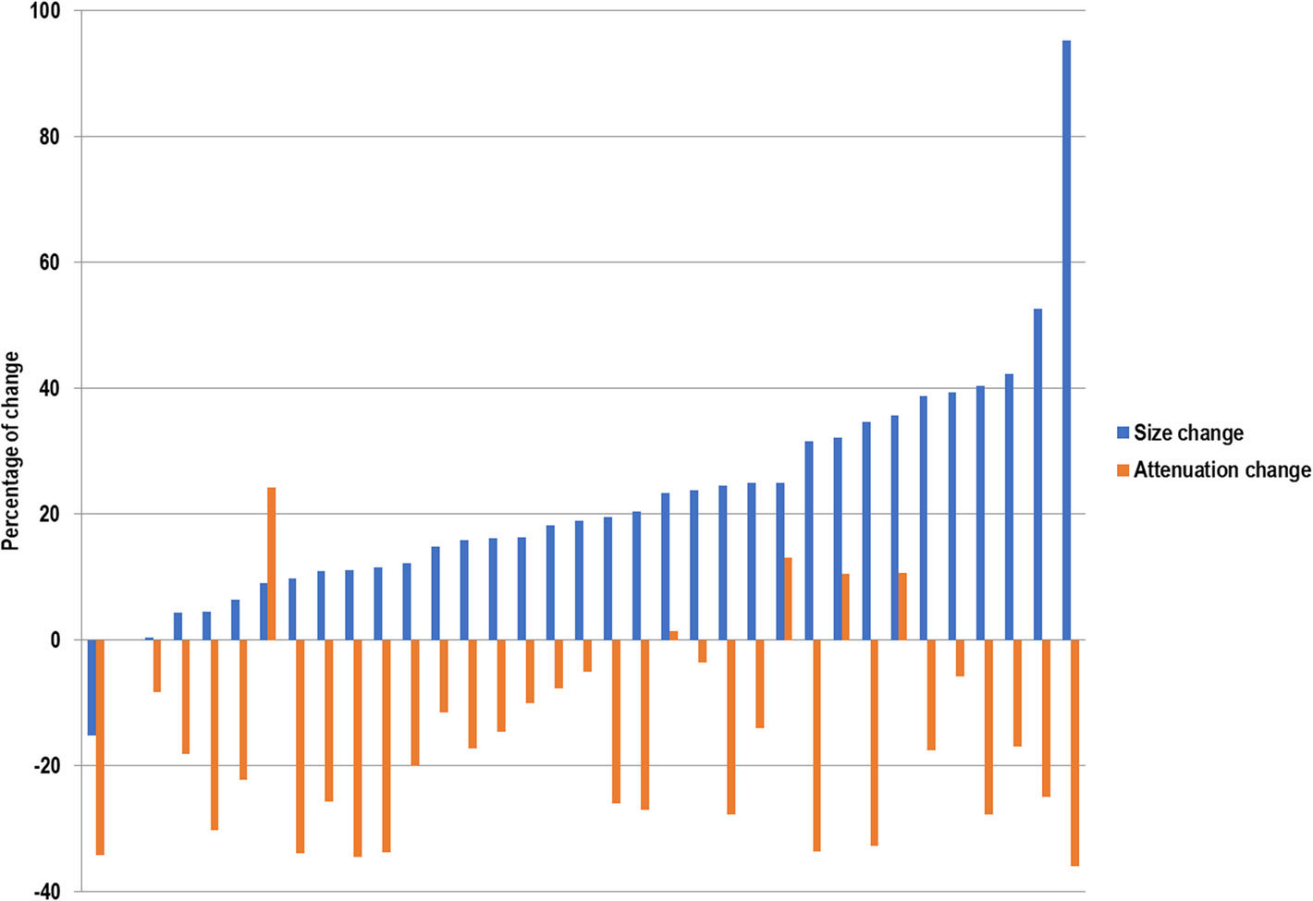
Waterfall plot summarizing the maximum percent change from baseline in the sum of longest diameter of target lesion and in attenuation at 2 months as measure by contrast-enhanced computed tomography

The variations of the imaging parameters (variation of the longest diameter according to RECIST1.1 and attenuation according to Choi criteria) of all the target lesions between baseline and 2 months are summarized in Fig. 2. There were no newly lesions identified. At 2 months, according to RECIST 1.1, there were no responders in the ITT2 population; 20 (57%) were SD (disease control rate) and 15 were PD (43%). At 2 months by CHOI criteria, 18 (51%) patients (95% CI 34–68.6%) were responders and 17 (48%) were non-responders (Table 3). At 2 months by mCHOI criteria, only one (3%) patient was responder and 34 (97%) were non responders. At 2 months by RECIST_10%_, one (3%) patient was responder, 19 (54%) were SD, and 16 (43%) were PD.

**Table 3:** Comparison between Choi and RECIST criteria at 2 months in the ITT2 population (*N* = 35)

	Responders (Choi)	Non-Responders (Choi)	Total
SD (RECIST 1.1)	11	9	20
PD (RECIST 1.1)	7	8	15
Total	18	17	35

**Fig. 3:**
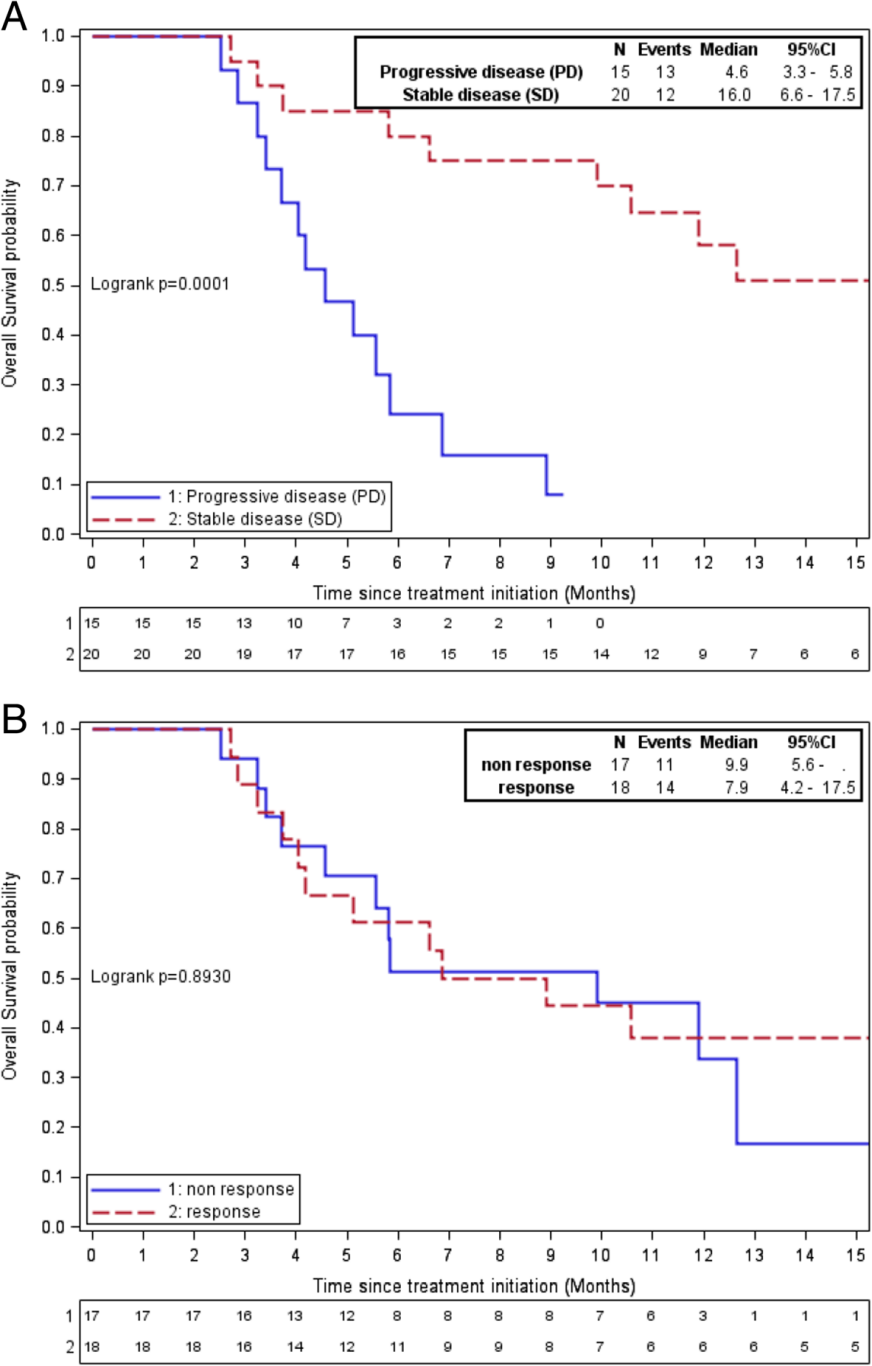
Overall survival as a function of (**a**) RECIST and (**b**) Choi criteria at 2 months in the ITT2 population

Median OS was 5.3 months (95% CI 3.7–8.6) In the ITT1 population and it was 8.9 months (95% CI 5.1–12.6) in the ITT2 population. In the ITT2 population, median OS was 16 months (95% CI 6.6–17.5) in SD patients at 2 months (*n* = 20) and 4.6 months (95% CI 3.3–5.8) in PD patients (*n* = 15), according to RECIST 1.1 (HR = 6.48, 95% CI 2.23–18.79; Fig. 3a). Median OS was 7.9 months (95% CI 4.2–17.5) in responders (*n* = 18) and was 9.9 months (95% CI 3.7-NA) in non-responders (*n* = 17) according to Choi (HR = 1.06, 95% CI 0.48–2.35 *p* = 0.89; Fig. 3b). Overall survival as a function of mChoi at 2 months was not calculated because all patients except one were classified as non-responders. Similarly, RECIST_10%_ did not differ significantly from RECIST 1.1 with only one patient having PR.

## Discussion

Multikinase inhibitors may reduce tumor attenuation due to decreased perfusion or metabolic activity with subsequent necrosis. The use of Choi criteria [2] allows a simple assessment of this phenomenon, even if an increase of size of the tumor is observed during treatment. Those criteria were successfully used to select GIST patients who will benefit from treatment with imatinib, mainly because the necrosis-induction effect of this biotherapy, which overcomes the intrinsic limitations of Choi criteria. Indeed, according to the Choi criteria, fast-growing untreated tumor with spontaneous central necrosis induced by locally insufficient angiogenesis, which leads to decreased enhancement of the central part of the lesion, would be expected to respond to treatment. This limitation might explain the contradictory results reported for different types of treatment for GIST or other tumor types, in which the drug-induced necrosis is less obvious than in case of imatinib therapy in patients with GIST [3]. Indeed, RECIST 1.1 and Choi criteria used to assess regorafenib activity in patients with advanced GIST after failure of imatinib and sunitinib showed similar clinical benefit rates [13]. Although the detection of response occurred sooner with Choi criteria, Choi criteria showed less concordance with OS than RECIST 1.1. These results had however retrospective nature and were based on the small number of patients. In our study, Choi criteria were less sensitive than those of RECIST1.1 criteria in predicting OS in mCRC patients treated by regorafenib.

An alternative Choi criteria (mChoi), in which a decrease of ≥15% in attenuation and of ≥10% in tumor size are require to define a responder have been proposed by Nathan et al. [6] in order to avoid the bias in assessing a fast-growing tumor with spontaneous necrosis. With mChoi criteria, the number of responders is expected to be lower compared to Choi criteria. Indeed, unlike Nathan et al. [6], who studied tyrosine kinase inhibitor (TKI), we found that mChoi criteria assigned all patients but one in the non-responder group and consequently was not discriminant at all. The difference could be probably explained by the fact that regorafenib induces few attenuation changes and almost no change of the size of the lesion. This might also explain why RECIST_10%_ and RECIST 1.1 showed very similar results.

Patients who showed benefit from regorafenib were considered SD according to RECIST1.1 and RECIST_10%._ All these patients except one exhibited a tumor growth rate between + 4.4% and + 20%. Hence, despite the fact that regorafenib could not stop the tumor burden to increase in size, patients could live longer when the percentage of size change was below 20% at 2 months.

## Conclusion

This study supports the postulate that SD at 2 months assessed with RECIST 1.1 represents true clinical benefit for mCRC patients treated with regorafenib. One of the major limitations of our study is the low patient sample size of the ITT2 population. However, this findings suggest that the simple assessment of attenuation change within the tumor using Choi criteria or mChoi criteria does not provide useful information about the efficacy of regorafenib in patients with mCRC.

## Data Availability

The datasets during and/or analysed during the current study available from the corresponding author on reasonable request.

## Abbreviations

AOI: Area of interest
CECT: Contrast-enhanced computed tomography
CI: Confidence interval
HR: Hazard ratio
HU: Hounsfield units
LD: Largest diameter
mChoi: Modified Choi criteria
mCRC: Metastatic colorectal carcinoma
ORR: Overall response rate
PFS: Progression-free survival
PR: Partial response
PS: Performance status
RECIST: Response evaluation criteria in solid tumors
ROI: Region of interest
SD: Standard deviation
TL: Target lesion
VOI: Volume of interest
